# A qualitative study of how COVID-19 impacts on Australians’ hopes and dreams

**DOI:** 10.1186/s12889-022-12746-4

**Published:** 2022-02-21

**Authors:** Quincy F. Huang, Floraidh Rolf, Lauren A. Booker, Taleah Moore, Sandra C. Thompson

**Affiliations:** 1 Western Australian Centre for Rural Health, University of Western Australia, PO Box 109, Geraldton, WA 6531 Australia; 2 School of Nursing and Health Studies, Georgetown University, Washington, DC USA; 3 Southern Queensland Rural Health, Faculty of Health and Behavioural Sciences, The University of Queensland, Charleville, Queensland Australia; 4 University Department of Rural Health, La Trobe Rural Health School, La Trobe University, Bendigo, Victoria Australia

**Keywords:** COVID-19, Travel, Australia, Goals, Pandemic, Outlook, Resilience, Fears, Aspirations

## Abstract

**Background:**

Although beginning in 2019, it was early in 2020 that the global community began to comprehend the significant impact that a pandemic of a new coronavirus might have on their own lives. This study was undertaken 6–9 months after significant public health restrictions were introduced within Australia and examined the impact of the COVID-19 on individuals’ hopes and dreams for their future.

**Methods:**

Community members who responded to a survey about COVID-19 were invited to participate in follow up interviews if they reported living with a chronic condition. Participants across Australia who consented were interviewed between August and December in 2020 over telephone or videoconferencing. A specific question was included regarding the impact of COVID-19 on their hopes and dreams for the future. Rapid identification of themes with an audio recordings technique was used to generate themes from the data.

**Results:**

The 90 participants were predominantly female (77%) and ranged in age from 20 to 81 years with a mean age of 50 years and lived in several Australian states. Following immersive analysis of interviews, the identified common themes impacting people’s hopes and dreams revealed: concerns for their own and others’ job stability and future work; the impact on travel both for holidays, business and reconnecting with family; reassessing of personal and social values; and the intergenerational impact of such a profound pandemic, with concern for younger people particularly prominent in those concerns. Participants reflected on their loss of future dreams, with possibilities they had planned and worked towards not possible in the short term.

**Conclusions:**

The responses provide a window into how people view their future goals and aspirations during a time of global and local instability and highlights the potential future impacts of the COVID-19 pandemic.

**Supplementary Information:**

The online version contains supplementary material available at 10.1186/s12889-022-12746-4.

## Background

Since the emergence of the COVID-19 pandemic in December of 2019, Australia has been proactive in controlling community transmission of the virus aiming for the goal of national control of pandemic impact. The first case of COVID-19 in Australia was diagnosed on 25 January 2020 [1]. As of 30 April 2021, Australia had a total of 29,801 cases of COVID-19 including 910 deaths [2]. Most cases (20,518) occurred in Victoria, followed by New South Wales (5477) and Queensland (1559). Most deaths due to COVID-19 have occurred in Victoria (820), New South Wales (54), and Tasmania (13) [2].

Over half (55%) of all COVID-19 cases have occurred in those aged 20 to 49 years, with the proportion of cases relatively similar for males and females. Most (94%) COVID-19 related deaths have occurred in individuals over the age of 70 years [2]. In comparison with the 150 million cases and over 3.1 million deaths from COVID-19 globally recorded on 30 April 2021, Australia makes up only a tiny proportion of both cases and deaths which is a testament to the effectiveness of the government’s mitigation plan for the virus, in addition to the advantage of the unique geography of Australia as an island nation at the bottom of the Southern Hemisphere [3]. The main pillars surrounding Australia’s COVID-19 pandemic response measures have been threefold: (1) surveillance, quarantine, and isolation, (2) testing and contact tracing, and (3) outbreak responsiveness [4]. The Australian government also closed the national borders and banned Australians from traveling overseas, a measure which was aimed at reducing transmission and infection [5].

While Australia saw benefits from the timely enactment of effective public health measures to control the spread of COVID-19, the pandemic’s effects on daily life of individuals and the communities in which they live have triggered profound changes. Emergency public health powers were enacted by state and territory governments in response to epidemiology and cases in the state at the time; restrictions on people’s liberties with respect to travel locally, within Australia and internationally, changed rapidly and so there were a patchwork of different arrangements at different times across Australia, with people expected to find up to date advice by visiting government websites (https://interstatequarantine.org.au/state-and-territory-border-closures/). While those in more densely populated cities had the greatest exposure to the virus, residents living in cross border areas were particularly impacted by the response to containing the virus [6]. People’s trajectory of the future and their long-term hope and dreams were impacted due to uncertainty and the restrictions imposed.

While pandemics are not a new phenomenon, this extraordinary time of global change in a connected digital age provides a distinctive context in which to examine the changes to people’s hopes and dreams during a major negative global shock such as the COVID-19 pandemic. The impact of pandemics on individuals’ hopes and dreams for the future is somewhat limited in research literature. Yet, whether it was the loss of a loved one, the loss of a job, or the loss of the sense of normalcy, people and communities experienced an impact on their lives because of the COVID-19 pandemic which warrants further understanding. Recognizing the impact, not just on the short term but on the long-term vision of people, is important to provide understanding and knowledge for future pandemic responses.

## Methods

This qualitative research project was conducted as part of a national collaborative effort spearheaded by a university department of rural health to collect data on individual and community experiences of the COVID-19 pandemic. The first phase of this project involved a survey of 677 people located across various states and territories in Australia, recruited through professional networks and social media. All survey members were asked if they would be willing to be contacted for a follow up interview and 416 responded positively. The original project was approved by the University of Queensland Human Research Ethics Committee (Approval number 2020000800) and amended for the qualitative approach with further reciprocal human ethics approvals were acquired from the investigators’ respective institutions before data collection and analysis. Out of the participants that expressed interest for a follow-up interview, 172 of those respondents identified themselves in the survey as living with a chronic illness. Details of the approach taken are available elsewhere [7]. Ninety of the original 172 participants across various Australian states ended up completing interviews, undertaken by phone by a team of researchers at institutions across Australia over 4 months (August to December 2020). Participants had to be 18 years or over, reside in Australia and have responded in the initial survey that they had a chronic illness.

Following carefully documented verbal informed consent, interviews were recorded, and field notes taken during the interviews. An interview guide was specifically developed for this research and is available as a Supplementary file (Interview Guide Attitudes and practices towards the COVID-19 pandemic in Australia. The interviews were semi-structured, with prompts centered on issues for exploration that included social distancing, handwashing, cleaning and disinfecting community behavior, leaving the house, government guidelines on social distancing, changes to daily life, vulnerability to COVID-19, impact on physical health, feeling safe, impact on mental health, risk of infection in one’s community. A specific question was included on how COVID-19 had affected their hopes and dreams for the future. While these themes served as a guide for interviews, investigators were encouraged to let the participants take control of the conversation to see what additional topics arose.

From the unique identifier in the data repository and using the demographic information collected during the initial survey, a participant number and identifier was constructed for each interview, linked with the unique identifier to support an audit trail. The interview identifier consisted of <Participant ID numbers _age _MM > in which the 90 interviews were labelled with a participant ID number from 1 to 90. The MM stands for Modified Monash [Model] and is a measure of rurality based on a model which measures remoteness and population size on a scale MM 1 to MM 7 where MM 1 is a major city and MM 7 is very remote.

The researchers used digital tagging to ascribe emergent themes and create a searchable dataset out of original field notes. The interviews asked specifically about participants’ hopes and dreams for the future and responses were reviewed to identify major themes common across multiple participants.

The authors used an inductive approach through immersion with the interview notes, return to and immersion in the digital recordings as needed, and establishment of links between codes to develop emergent themes, discussion of connections to identify the main themes on hopes and dreams for the future aggregated across multiple interviews. Further discussion occurred amongst the five researchers who then reviewed the relevant notes and interview recordings in greater detail.

## Results

Participants were predominantly female (77%), and individuals ranged in age from 20 to 81 years with a mean age of 50 years. The interviews covered many aspects of participants’ psychosocial wellbeing including dealing with the uncertainly of the future with participants commenting on a range of issues relevant to their future wellbeing. Four main themes emerged from the data specific to hopes and dreams for the future: job stability, travel, reassessing values, and generational impact.

### Job stability

For some young adults, the COVID-19 pandemic in Australia led to the loss of education and job opportunities which ultimately impacted on their hopes and dreams for the future (Walsh, 2020). Adults aged 35 years and older also experienced job insecurity, with unemployment rates rising 14.8% in the period between March and September 2020 [8]. Job stability was a common theme among interview participants, with the main concerns being losing a job and not being able to find a job in the future. A similar proportion of males and females expressed concern for their job security, and this worry was felt across all age groups. While concerns for future job security appeared widely felt at the time, whether these fears persist in the short- or long-term remains to be seen and are likely to depend on both individual and larger macro system factors. This sentiment was captured by this woman on searching for a job: “*the chances of finding a job are always fraught, but the [current] economic situation is going to make that so much more difficult*” (Participant 63_F_46_2), while an individual who was employed commented, *“[the pandemic] is probably going to lose me my job*” (Participant 65_M_65_5). Depending on their type of work, some participants felt the loss of job security more acutely, *“[Musicians] playing four, five nights a week is their livelihood and a big part of their income*” (Participant 3_F_49_2).

The shift to a virtual environment also impacted students’ plans and necessitated changes to accommodate a new reality. One student commented, “*I played around with my majors to make sure that I am going to be able to work from home and have more flexible working conditions*” (Participant 39_F_34_5). Young people expressed their concerns regarding education and what the future held in terms of jobs, wondering whether education would lead to a better job. As one student commented “*The risk of me having no work is that I probably will continue to study and may end up with a postgrad and in more debt for something I am not completely interested in*” (Participant 49_F_31_1). Even those with a clear future career pathway felt less confident about what lay ahead, for example a medical student recounted a fear of readiness for graduation and practice after a long break from patient contact and clinical placement. Such concerns were not unique to students with apprehension around job security extending to those currently employed and retirees. Individuals not currently in school also remarked on going back to school being a possibility if jobs were unavailable. Teachers and educational faculty members also felt the strain the pandemic put on their careers. As one academic commented, “*We’re facing a very real possibility that there will be [no jobs] to apply for, fellowships will be gone, funding to universities will drop, and the recovery for something like that is very long*” (Participant 25_F_37_1).

Concerns were also expressed surrounding the economic insecurity that resulted from loss of jobs which were recognized as disproportionately affecting some people: “*we’ve got a huge portion of the population that can’t even look after themselves if they miss a paycheck*” (Participant 53_M_36_2). Economic uncertainty and security of employment were very real concerns for many who were unemployed or unsure what their jobs would look like in the future. Even those who were employed voiced considerable apprehension for the future; “*I’m still concerned for my own work; I am not sure what the future is going to look like for my work. I hope that it looks ok and the skills that we have…are going to be supported*” (Participant 40_F_42_3).

Despite the overwhelming fears and concerns, many individuals found the changing landscape of in-person work moving to a virtual environment to be a positive thing and to be associated with other benefits: “*Working from home is not an impossible dream, and with the population pressures that we are under it would be nice if we could keep large populations working from home and put less pressure on public transportation and nature itself*” (Participant 16_F_64_5).

Of the 90 individuals interviewed, at least a quarter of them raised job security in their discussion of hopes and dreams for the future, with concerns raised equally by men and women. The age range for participants who mentioned job security was 20 years to 66 years, with the mean age being 48 years, showing that concern for job security was felt across a wide variety of job-participating age groups.

### Travel

The ability to travel both domestically and internationally was greatly hindered by border closures instituted by both state and federal governments. Beginning in late March 2020, Australian borders were closed to everyone except for Australian citizens, residents, and immediate family members [9].

Uncertainty surrounding travel, frustration of cancelled plans and not being able to plan overseas trips or seeing family and friends in the future were common preoccupations for many participants. A recent retiree commented, “*COVID has made me rethink travel, I am not game enough to go overseas for a while… if we do end up travelling it will be less planned, more spontaneous*” (Participant_47_F_60_1) Similarly, another individual discussed how he was “*reluctant to plan any holidays for fear that they will be cancelled*” (Participant_89_F_56_2).

Personal travel was not the only thing impacted by the border closures. One individual encapsulated the concerns many felt when he expressed, “*I don’t know how Australia will come out of it… I don’t know what it means for future travel of products and exports and who can afford to travel*” (Participant_41_F_M_36_2). He was concerned with the movement of goods and services through the wider global economy, which was brought on by the drastic change in travel and border closures.

The impact on individual travel goals was not limited to international travel. Domestic border closures and restrictions were announced in the Northern Territory, Queensland, South Australia, Tasmania, and Western Australia, which made it difficult or impossible for individuals to travel to or from these states [10]. While these restrictions were implemented to reduce the community transmission of COVID-19 in the present, the fear of future travel restrictions being implemented is one that may persist beyond the pandemic. A woman who was an avid domestic traveler before the pandemic “*cancelled caravanning around Western Australia due to border restrictions and not feeling safe of people around you*” (Participant 47_F_60_1). In contrast, others rethought their plans for travel to keep them much closer to home, as illustrated by an individual who had decided her *“future travel will be in Australia instead*” (Participant 11_F_65_2).

In addition to the change in travel plans, travel restrictions also meant missing out on family milestones for those who had relatives in different areas. An individual “*missed two weddings and a funeral in the last two months [because he] has not been able to leave the country*” (Participant 41_M_36_2). This sentiment was shared by another who said, “*we miss that in-person contact with people that you are close to…my husband’s family lives in New Zealand and he usually travels over to see them every year*” (Participant 42_F_55_2).

Travel was one of the main aspects of individuals’ future hopes and dreams that were negatively impacted by the pandemic. Out of the 90 individuals interviewed, over half mentioned future travel in their discussions of hopes and dreams, and it was more commonly mentioned by women than men and across a wide range of age groups, from 23 to 75 years.

### Reassessing values

The COVID-19 pandemic changed the day-to-day lives of people all over the world in profound and drastic ways. In Australia, daily commutes and social interactions were limited, and people found themselves staying at home and spending time with smaller social circles. People from diverse points along the adult lifespan shared thoughts around making each day count and realizing the value of family above material wealth. For many participants, this change meant slowing down and appreciating the present as much as the future, “*it has made me think more about making today count*” (Participant 33_F_56_6). This sentiment was expressed by many others, including a woman who commented, “*material things are not as important as having your family and living in a [healthy] community*” (Participant 9_F_36_6).

In addition to a greater appreciation for physical health, participants made repeated mentions of improved environmental health. The positive effect the pandemic had on climate change is a theme that several participants mentioned as something they would like to see continue after the pandemic. One woman encapsulated the views of many when she observed, “*the forced lockdowns have forced the world to change, and we can see the effects of it [on the environment]*” (Participant 33_F_56_6). As another participant similarly noted “*environmentally there has been good benefits from people who have not been able to travel… hopefully it has made people a bit more conscious [of] how their lifestyle does impact the environment*” (Participant 90_F_49_2). The positive effect of fewer commuters was also noted by younger participants “*As a society we are going to be able to reduce greenhouse gas emissions by people working from home*” (Participant 39_F_34_5).

Reassessing values in occurred commonly in consideration of future hopes and dreams, and was raised more by women than men, again across a broad age range.

### Generational impact

While the COVID-19 pandemic personally affected people across all age groups, another common theme across many interviews was their concern for the younger generations, with 11 women and 7 men mentioning the future impact on younger generations in their discussions about hopes and dreams. More males than females expressed concerns for younger generations, and this sentiment was predominantly found in the older age groups. This generational concern spanned issues from economic impact to mental impact and were expressed by concerned parents and grandparents. Fear for the national economy was consistently expressed when discussing generational impacts, regardless of participants having children or being child-free. Some participants who were also mothers talked about the burden of their children ultimately paying for government welfare initiatives such as unemployment assistance like JobKeeper and JobSeeker, or the impact on their children’s dreams and their employment future. Yet, child-free participants also expressed concern at generational economic burden. Perceptions of a slow recovery and long-term economic effects transcended individual impact and were positioned by many participants as a community encumbrance.

Adults without children still recognized the economic impact that would be felt in the years to come. As observed by one male, “*there are going to be long-term economic effects but not really affecting me*” (Participant 65_M_65_5), a sentiment also noted by another similarly aged woman, “*we are not going to get over what’s happened to the economy anytime soon*” (Participant_84_F_64_5).

Furthermore, mental health concerns for younger populations were noted frequently. A mother mentioned the struggles she saw in younger generations, stating, “*it’s been quite hard for young people, the resilience is disappearing from them very quickly and I think we as adults in the community are partly responsible for that*” (Participant 44_F_55_7).

The overall worry for younger generations was one that is widely recognized by Australians as they looked forward towards recovery and moving forward. From loss of jobs to heightened national debt, the impact the pandemic had on Australia and globally was recognized as one that would be felt for years to come. As one mother expressed, “*as a parent, when thinking about my hopes and dreams for the future, it is going to be the impact on my children’s hopes and dreams*” (Participant_29_F_45_1).

## Discussion

The pandemic created a unique situation in which people experienced a sense of powerlessness, both at the hands of the virus and at the interventions imposed by state and federal government. Individuals were limited in their capacity to change their current situation, with lack of agency known to be a contributor to stress and hopelessness [11]. Issues related to psychosocial wellbeing were evident throughout the interviews with interviewees referring to their mental health but also considering new possibilities for the future. Considerable attention has already been given to the mental health consequences of COVID-19 across a variety of different age groups [12, 13].

‘Hopes and dreams’ was a term used to describe a participant’s outlook on the future, the desire of an improved situation (which may be about personal, professional, or other aspects of an individual’s existence), the possible fear or concern of a less-promising future, and both practical and aspirational objectives to strive for. Although ‘hopes and dreams’ are not typically qualities that may be empirically measured (that is, comparing one person’s hopes and dreams to another person’s does not validate or invalidate that of one person over another), it is reasonable to recognize that each person considers their personal hopes and dreams paramount to their individual value systems. As the participants described their feelings, optimism or concerns on their individual hopes and dreams, they were stating and sharing their assessment of their current times living during a pandemic, their predictions for the future following the pandemic, and – importantly – what their place may or may not be in the post-pandemic world.

This study was undertaken about 6–8 months after COVID-19 first reached Australia and reflects the concerns and aspirations of people at that time. Psychosocial wellbeing, job security, travel, reassessing values, and generational impact were the focal themes expressed repeatedly by participants, spanning age and sex. While individual participants recounted their own hopes and concerns for the future, these themes dominated the interviews.

Research surrounding the psychological impacts of the COVID-19 pandemic suggests that many people experience feelings of grief due to loss of loved ones, loss of a sense of normalcy, or both [14]. However, this study found that even though individuals were limited in their capacity to change their current situation, many remained optimistic with respect to their hopes and dreams for the long term.

The concern of job security was not felt equally among all individuals, depending on personal socioeconomic factors. In Australia, 13.6% of the population lives below the poverty line, a higher rate than many other Organization for Economic Cooperation and Development (OECD) countries [15] and had detrimental impacts on economies all over the world [16]. From February 2020 to August 2020, arts and recreation services employment in Australia decreased by 17.9% [8]. This was the second highest decrease in employment for any industry in Australia during the pandemic, with the highest being accommodation and food services jobs which decreased by 18.2% [8]. Individuals who were not deemed essential workers and who did not have the ability to work from home were in an especially difficult situation. By the end of 2020, 6.5% of Australians were unemployed, the highest rate since 2001 [17]. Young adults aged 15 to 34 years accounted for 57% of individuals unemployed in Australia from March 2020 to September 2020 [8]. This high level of unemployment combined with a tumultuous job market set the background for fears of limited or lack of job stability in the future.

While job concerns appeared nearly universal, these concerns were not equally experienced. By switching to a virtual environment some people found flexibility, reduced commuting, and the additional time with family that work from home allowed was an aspect of the pandemic that many wanted to see retained in the future. While some were pleased to be protected within closed borders, for others this caused major inconvenience and challenges related to the separation from loved ones and their usual way of life [6].

In addition to job concerns, travel was another major way of Australian life impacted by the pandemic. As an island nation, Australian citizens frequently travelled overseas, with the top destinations being New Zealand, Indonesia, and the United States [18]. International arrivals into Australia decreased 81% in 2020 as compared to 2019, with international travel receipts decreasing by 43% [19]. In addition to the restrictions for incoming travelers, there was a ban on overseas travel from Australia; although as of March 2021 this was amended to allow for travel to and from New Zealand [9]. Travel restrictions have affected trade and heightened risk around Australia’s economic growth and prosperity, given the country relies heavily on exports and imports. In 2019, Australia imported USD (United States Dollars) 209 billion worth of products and exported USD 284 billion worth of products [20]. While there have been no specific restrictions on imports due to the COVID-19 pandemic, there have been significant delays because of travel restrictions [21]. Australians’ perceptions of future travel were generally pessimistic, with many saying they would not travel overseas for quite a while. Some individuals saw a greater opportunity to travel domestically within Australia, but still expressed health concerns and worry crossing state lines. Females accounted for a higher proportion of individuals who expressed future concerns surrounding travel, and this fear was found most heavily in older age groups, predominantly among retirees who had planned on traveling more regularly. The shock of the pandemic and its associated restrictions and changes in everyday life, while hearing and seeing stories of high mortality elsewhere catalyzed many to reassess their values. It has been argued that there has been a psychological shift from an individualist mindset to a collectivist mindset because of the pandemic, with communities stepping up to provide support where needed [22]. Whether this reassessment of values will last post-COVID remains to be seen; however, research suggests that humans will adapt back to the world returning to its commercialized environment, the same way they adapted to the temporary shut-down of businesses and schools [22]. Within the theme of reassessing values, care for the environment was also repeatedly mentioned. The pandemic arrived in Australia shortly after a season of devastating bushfires which arose after a period of drought and had severely impacted wildlife and people, destroying many hectares of bushland, rural properties as well as homes and infrastructure [23]. People were awake to the problems of climate change and the need to do something. Because of the impact of COVID-19 on transport and industry, both in Australia and around the world there were notable positive environmental changes. Air pollution and greenhouse gas emissions were greatly reduced, with nitrogen dioxide (NO_2_) and particulate matter (PM_2.5_) levels lowering worldwide [24]. Additionally, noise pollution reduction and the ecological restoration of tourist hotspots made it possible for the natural ecology to temporarily recover [24]. The greater sense of community and care for the natural world were values participants verbalized in their interviews when discussing their hopes and dreams for the future. A higher proportion of females than males mentioned reassessing values, and around one-fifth of respondents discussed this theme of reassessing values.

The final theme, generational impact stood out when examining participant outlook. Generational impact primarily surrounded economic concerns. In response to the national shutdown, the Australian government supported its citizens through tax breaks, wage subsidies, and cash payments to the most vulnerable populations [25]. Federal government budget forecasting anticipated that Australia’s national debt could reach close to AUD (Australian Dollars) 1 trillion, the burden of which would primarily be felt among younger workers (Worthington, 2020). The disproportionate burden on the younger generation who do not have established financial security in in helping to rebuild Australia’s economy was the main reason behind concerns for this group. The other aspect of generational impact surrounded concerns for the mental wellbeing of younger generations. A 2020 survey found that younger generations are showing the most resilience during the COVID-19 pandemic and are committed to contributing positively to their communities and the entire world [26]. Millennials and Generation Z are defined as individuals born between 1981 and 2012 [27]. While this survey may be an indicator of the overall resilience and motivation of younger generations, there are still many concerned for the mental health of this younger age group because of the pandemic.

While the four major themes of job security, travel, reassessing values, and generational impact reflect the ways in which the COVID-19 pandemic impacted hopes and dreams for the future in both an optimistic and pessimistic way, the resilience that individuals believed themselves to exhibit during this trying time was the true indicator of the level of hope they were able to maintain for their futures.

### Limitations

This study was conducted at the beginning of the pandemic and provides a good insight into the sentiments of a variety Australians at the early stages of COVID-19. It included a diversity of interviews but we acknowledge several limitations. The data collection occurred over a few months, effectively representing a one point in time. Even though the geographic coverage was broad, numbers were skewed to participants from Queensland. In addition, as common with much population-focused qualitative research, there were more female participants. Living with chronic illness was noted by the invited participants in their initial survey responses, but many did not identify the illness itself. During the interview some participants made no mention of their chronic illness, and a few indicated they did not have a chronic illness, did not consider their illness to be relevant to their experience of the pandemic, or did not disclose this information to the interviewer. Hence, no analysis was undertaken of the impact of a chronic disease on participants’ hopes and dreams for the future.

## Conclusions

A major global event, such as the COVID-19 pandemic, has provided a window into how people view their future goals and aspirations during this time of worldwide and local instability. Initially, people were asked about their own hopes and dreams for the future, but what emerged were broader concerns about the economy, generational and travel/ border closure impacts. The exploration of such experiences provides insight and knowledge into the long-term impacts of the pandemic which will help guide future preparation and management, both for the individual and the wider community. Also, this study highlights that during a global pandemic, people’s perception of their future, while negative at times, shows hope and resilience. While hopes and dreams for the future are unique to each person, certain themes appeared commonly during the pandemic and were felt across the population, regardless of age and gender.

## Supplementary Information

**Additional file 1.** Interview Guide_Attitudes and practices towards the COVID-19 pandemic.

## Data Availability

The data that supports the findings of this study are available from the corresponding author [ST] upon reasonable request.

## Abbreviations

AUD: Australian Dollars
F: Female
M: Male
MM: Monash Modified
OECD: Organization for Economic Cooperation and Development
USD: United States Dollars
WA: Western Australia
