# Supplementary material for: A qualitative study of how COVID-19 impacts on Australians’ hopes and dreams
Source: BMC Public Health. 2022 Feb 21;22:367. doi: 10.1186/s12889-022-12746-4 (PMC8860267; doi:10.1186/s12889-022-12746-4)
Supplement: Supplementary file 1 — Additional file 1. Interview Guide_Attitudes and practices towards the COVID-19 pandemic. [file 12889_2022_12746_MOESM1_ESM.docx]

**ATTITUDES AND PRACTICES TOWARDS THE COVID-19 PANDEMIC IN AUSTRALIA:**

**INTERVIEW QUESTIONS**

**Interview notes**

• Interviews are to be conducted via phone, face-to-face or video conference (i.e. Zoom).

• The following questions are an indicative list of what may be asked during these interviews.

• The specific interview questions for the follow-up interviews will be guided by the responses of the online survey. The research team is aware that an ethics amendment will be sought if interview questions are changed or added.

**Before the interview**

• Ensure verbal consent is received for the interview.

• Explain that the purpose of the interview is to further explore attitudes and practices relating to the COVID-19 pandemic.

• Explain that the interview will last for approximately 30 minutes.

• Explain that the interview will be recorded to allow for accurate transcription.

• Explain that participation in the interview is voluntary and the interview can be stopped at any time.

• Explain that any identifiable data (such as the participant’s name) will be removed prior to data analysis.

**Question guide**

*1. Can you tell me about your experiences of life during the COVID-19 pandemic?*

*2. What are your thoughts about the Australian Government’s social distancing and hygiene guidelines? Can you provide examples of how they affected your everyday life?*

*3. Do you believe the Australian Government’s social distancing guidelines have been appropriate for your community? If not, why?*

*4. What changes have you made/did you make in your everyday life to reduce your risk of infection? Will/have you continue/d these practices after the restrictions are/were lifted? Is there anything you feel you would continue to do in the future after the pandemic has passed? and why?*

*5. Do you believe there are other things that could be done/could have been done in your community in response to the COVID-19 pandemic?*

*6. How has everyday life for you, your, friends, family and your community changed as a result of the COVID-19 pandemic?*

*7. Do you think that positives have emerged from the COVID-19 epidemic?, If so, what are they?*

*8. How has COVID-19 impacted upon your hopes and dreams for the future?*

**Additional Probing Questions (as needed):**

• *Could you explain that a little bit more?*

• *Could you give an example?*

• *Do you think others would feel the same way?*

• *How could this be better achieved?*

• *Why do you feel that is the case?*

• *Would you add anything else?*
